# Feeling like the enemy: the emotion management and alienation of hospital doctors

**DOI:** 10.3389/fsoc.2023.1232555

**Published:** 2023-08-24

**Authors:** John-Paul Byrne, Jennifer Creese, Robert McMurray, Richard W. Costello, Anne Matthews, Niamh Humphries

**Affiliations:** ^1^Graduate School of Healthcare Management, RCSI University of Medicine and Health Sciences, Dublin, Ireland; ^2^Department of Population Health Sciences, University of Leicester, Leicester, United Kingdom; ^3^Department of Medicine, RCSI University of Medicine and Health Sciences, Dublin, Ireland; ^4^School of Nursing, Psychotherapy and Community Health, Dublin City University, Dublin, Ireland

**Keywords:** emotion management, alienation, hospital doctors, ethnography, research methodology, Ireland, sociology of health, sociology of emotion

## Abstract

**Introduction:**

Globally, an epidemic of psychological distress, burnout, and workforce attrition signify an acute deterioration in hospital doctors' relationship with their work—intensified by COVID-19. This deterioration is more complicated than individual responses to workplace stress, as it is heavily regulated by social, professional, and organizational structures. Moving past burnout as a discrete “outcome,” we draw on theories of emotion management and alienation to analyze the strategies through which hospital doctors *continue to provide care* in the face of resource-constraints and psychological strain.

**Methods:**

We used Mobile Instant Messaging Ethnography (MIME), a novel form of remote ethnography comprising a long-term exchange of digital messages to elicit “live” reflections on work-life experiences and feelings.

**Results:**

The results delineate two primary emotion-management strategies—acquiescence and depersonalization—used by the hospital doctors to suppress negative feelings and emotions (e.g., anger, frustration, and guilt) stemming from the disconnect between professional norms of expertise and self-sacrifice, and organizational realities of impotence and self-preservation.

**Discussion:**

Illustrating the continued relevant of alienation, extending its application to doctors who disconnect to survive, we show how the socio-cultural ideals of the medical profession (expertise and self-sacrifice) are experienced through the emotion-management and self-estrangement of hospital doctors. Practically, the deterioration of hospital doctors' relationship with work is a threat to health systems and organizations. The paper highlights the importance of understanding the social structures and disconnects that shape this deteriorating relationship and the broad futility of self-care interventions embedded in work contexts of unrealized professional ideals, organizational resource deficits and unhappy doctors, patients, and families.

## Introduction

*AS A MEMBER OF THE MEDICAL PROFESSION:*

*I SOLEMNLY PLEDGE to dedicate my life to the service of humanity…*.

*I WILL FOSTER the honor and noble traditions of the medical profession…*.

*I WILL SHARE my medical knowledge for the benefit of the patient and the advancement of healthcare;*

*I WILL ATTEND TO my own health, wellbeing, and abilities in order to provide care of the highest standard…*

- World Medical Association (2017). The Physician's Pledge of the Declaration of Geneva [caps in original].

*Key concept, said the Fat Man, to think that you're doing a shitty job. If you resign yourself to doing a shitty job, you go ahead and get the job done…*

- Shem (1978, p. 75). *The House of God*.

Medicine is a “profession in retreat” (Zuger, 2004), plagued by a widening gap between expectations and reality, which shapes not only how doctors feel about their work but the impact it has on their satisfaction and wellbeing (Zuger, 2004; Gerada, 2021; McKinlay and Marceau, 2011; O'Mahony, 2019). Gerada (2021), drawing on her experience of providing mental health services to doctors (“the doctor's doctor”), presents a detailed overview of the chronic stressors and emotional hazards of modern-day medicine. She highlights the enduring and powerful impact of professional norms which are inculcated through the hidden curriculum of medical training (Becker et al., 1961; Scambler, 1991) so that medicine becomes “…more than just something I *do*, but something I *am*” (Gerada, 2021, p. xii). Acknowledging the power of this identity, Gerada (2021) also notes how it is often inscribed by guilt, expertise, suffering, “the rules of self-sacrifice,” and the requirement to manage often-unrealistic patient and self-expectations in the face of health system resource-deficits.

The Declaration of Geneva represents the contemporary Hippocratic Oath, adopted by the World Medical Association (WMA) in 1948, which takes the form of a physician's pledge delineating the duties and ethical principles of the global medical profession (Parsa-Parsi, 2017). To illustrate the norms of self-sacrifice in medicine, Gerada (2021) notes the pertinence of the first line of the Declaration of Geneva where doctors pledge their lives to service. When discussing the need to quell perfectionist self-expectations with learned coping mechanisms, Gerada (2021) turns to Samuel Shem's (1978) fictional satire of an intern's 1st year in an American hospital where the senior doctor advises his junior colleague to accept lower standards to get the job done. We include and adjoin these statements here, adding more lines from the Declaration of Geneva, for two reasons. Firstly, the diverging sentiments encapsulate the struggle between expectations and reality at the heart of medicine, as what is pledged and achieved are often misaligned and require emotion management strategies. Secondly, they point to the complex links between expectations, norms, and practices that comprise doctor's relationship with their profession. Interestingly, the last two sentences included in the extract from the Declaration of Geneva are additions from the 2017 revisions, indicating a current emphasis on doctors sharing medical expertise and attending to their own wellbeing to ensure the highest quality care. Parsa-Parsi (2017, p. 1972) notes how a focus on wellbeing points to “the humanity of physicians” and the role “self-care can play in improving patient care.”

The introductory statements indicate the complex social structures and professional norms underpinning doctors' relationship with their work. This relationship is one which involves more than just a code of ethics or socio-cultural ideals (Scambler, 1991), but appears to be undergoing an acute deterioration signified by high rates of psychological distress, burnout and workforce attrition (West et al., 2016; Shanafelt and Noseworthy, 2017; Humphries et al., 2019). Taking this deteriorating relationship as a starting point, we analyze the emotion-management strategies through which hospital doctors *continue to provide care* in the face of severe resource-constraints and alienating conditions—with considerable implications for doctor wellbeing and patient care.

### A profession in retreat: from power to burnout

Medicine has historically been seen as a profession of power, prestige, specialized expertise and personal sacrifice (Scambler, 1991; Jenkins, 2020; Gerada, 2021). However, the modern translation of this position has been one of system-resource deficits, extreme working patterns and psychological strain (Humphries et al., 2019; Byrne et al., 2021; Creese et al., 2021). Gerada (2021), noting the contrast to previous medical generations, notes that hospital doctors often have no place to put their coat, no place to get some rest on long calls, and limited food, often relying on vending machines for sustenance while at work. Related to this, the latest challenge—indicated by the 2017 addition of a pledge related to doctor's wellbeing in the Declaration of Geneva—is burnout.

Burnout refers to an interplay of chronic work-related stressors resulting in feelings of exhaustion, cynicism or detachment, and a sense of ineffectiveness or lack of accomplishment (Maslach and Leiter, 2016). Even prior to the COVID-19 pandemic, burnout had become a significant issue for the medical profession globally. In the US, West et al. (2016) described burnout as reaching “epidemic” levels, while a 2019 report from National Academies of Sciences Engineering and Medicine (2019) depict it as an urgent public health issue negatively impacting clinicians, patients, trainees, and healthcare organizations (National Academies of Sciences Engineering and Medicine, 2019). In Ireland, Hayes et al. (2019) found almost one-in-three doctors met the criteria for burnout. In terms of impact, research has shown associations between burnout and increased patient morbidity and safety errors, decreased patient satisfaction, increased financial costs, and poor workforce wellbeing and attrition (West et al., 2016; Shanafelt and Noseworthy, 2017; Han et al., 2019). Despite having well-validated measures (e.g., Maslach Burnout Inventory—MBI, Maslach and Jackson, 1981) and being studied across a range of professions, the conceptualization and definition of burnout remains a topic of debate with relatively little consensus on a full understanding (Schwenk and Gold, 2018).

Recent approaches to burnout have called for more attention to the role of organizational and professional contexts in generating and sustaining chronic workplace stressors (Montgomery et al., 2019; WHO, 2019). However, Schaufeli (2021, p. 170) notes the absence of a consensus on burnout's “enigmatic” antecedents and consequences. Much debate revolves around the common practice of equating burnout solely with exhaustion (Leiter and Maslach, 2016; Schwenk and Gold, 2018). Critiquing this equivalency, Leiter and Maslach (2016) state that individuals experiencing burnout are not just tired, they are *alienated* through some form of crisis in meaning, values and feelings. They also note the relative neglect of cynicism and inefficacy in burnout research—despite being central features of their early conceptualization (Leiter and Maslach, 2016). Schaufeli (2021) adds to this debate by highlighting approaches that deem cynicism and detachment as a coping mechanism for exhaustion, and inefficacy a consequence. Alternatively, describing the frontline experience of burnout for one NHS doctor during the first wave of COVID-19, Chaudhry et al. (2021) posit professional efficacy as part of the *process* of burnout rather than an *outcome*. For this doctor, maintenance of professional efficacy was the beginning of her experience of burnout with depersonalization employed as a coping strategy to deal with the intensity of organizational demands. Supporting Montgomery et al. (2019) and Panagioti et al. (2017), these findings point to an “extra-organizational” influence on the burnout experience including national policy, perception of government support and public opinion (Chaudhry et al., 2021). Burnout has become a “knot” (Schaufeli, 2021) which requires unraveling through novel forms of research and theorizing—especially regarding the experience of cynicism and inefficacy (Leiter and Maslach, 2016).

The “burnout experience”—however conceived—is much more complicated than an individual response to chronic workplace stressors. It is heavily regulated by professional, organizational, and societal influences. Most burnout research draws on data from quantitative research instruments (e.g., MBI) which seek to measure, analyze, and aggregate individuals' feelings according to de-contextualized variables (Hayes et al., 2019). The MBI is a survey of how an individual feels about their work at a point in time according to theoretically and empirically validated dimensions (Maslach and Jackson, 1981). While this is relevant from a prevalence perspective, it points to a mismatch between new burnout research approaches that seek to bring in the role of organizational context and system (Panagioti et al., 2017; Montgomery et al., 2019), and data essentially founded on discrete individual responses. At its heart, burnout refers to a deterioration in an individual's relationship with their work—a relationship that is shaped by a range of social structures and professional norms that affect everyday emotions and behaviors. To unravel this “knot,” we turn to a more sociologically adept concept*, alienation*. Framing doctors' work-self-wellbeing relationship in terms of alienation enables a wider analytical frame that encompasses both social-psychological processes (Seeman, 1959) and relational and structural conditions (Twining, 1980; Soffia et al., 2022) and the impact these have on everyday processes, experiences and feelings.

### Alienation: bridging structures and emotions

Alienation represents one of sociology's enduring conceptual tools for understanding the relationship between work and the self. From Marx' original conceptualization (Marx, 1964) through Blauner's (1964) analysis of industrial technology and freedom to modern accounts of relational conditions like voice and overload (Conway et al., 2020) and meaningfulness of work (Soffia et al., 2022) and their impact on wellbeing, alienation continues to appeal; “…standing as it does at the intersection of social-structural conditions and psychological orientation…” (Kohn, 1976, p. 111). Thus, alienation has been linked to a range of social-psychological consequences including meaninglessness, powerlessness, emotional exhaustion, isolation, and self-estrangement (Seeman, 1959; Blauner, 1964; Twining, 1980; Maslach and Jackson, 1981; Conway et al., 2020). Marx (1964) defined four factors of alienated labor—separation from the product of labor, separation from the process of labor, separation from natural conscious activity and essence (“species being”), and separation from other workers. Key here is the focus on the social relations underpinning how and why work is done, the level of control workers have over their labor, and opportunities for self-realization (Soffia et al., 2022). Post-industrial accounts of alienation continue to emphasize the effect of the externalization of autonomous work and modern forms of constrained agency.

Rosa (2015) theorizes a loss of control due to the acceleration of modern society powered by three mutually reinforcing forms of acceleration: technical, social change, and pace of life. Berardi (2009) points to an autonomous form of alienation for the “cognitariat”—a class of workers, whose labor is cognitive, yet still see their working conditions powerfully shaped by external sources via the synchronizing of professional norms, techno-economic structures, and economic markets. Rosa (2015) and Berardi (2009) point to a modern form of alienation where workers may have high levels of workplace autonomy (unlike industrial work) *and* a sense of estrangement, as well as constrained opportunities to utilize their skills to affirm their sense of self (Soffia et al., 2022). For doctors, work demands and norms are profoundly shaped by the social structures of medical training, professional norms and status hierarchies, regulatory and administrative demands, and organizational deficits (Becker et al., 1961; Jenkins, 2020; Byrne et al., 2021; Gerada, 2021). Zuger (2004) notes how much of doctors' dissatisfaction with medical practice is founded on a series of discrepancies including between what patients expect and what doctors can provide, and between the standards learned in medical training and the “compromises forced by practice.” Building on Braithwaite et al.'s (2017) distinction between work-as-imagined and work-as-done in relation to health system improvement, Zuger's (2004) discrepancies point to a mismatch between a medicine that is professionally imagined and a medicine that is organizationally experienced and enacted. Gerada (2021) argues that this mismatch between the expectations of medicine and its organizational reality fuels mental health issues for doctors. One such stressor has been conceptualized as moral injury—where doctors' commit, witness, or fail to prevent acts that contravene their deeply held beliefs and morals (Dean et al., 2019). Nonetheless, the core themes running throughout alienation's journey from industrial to post-industrial work contexts are externalization, disconnect, loss of agency, and estrangement. This range of features points to both the richness of alienation as a concept, and the potential for ambiguity due to its wide application (Nair and Vohra, 2012).

Debate around the conceptualization of alienation has revolved around three points: emphasis on structural or social-psychological conditions, uni-dimensionality vs. multi-dimensionality, and the overlap between antecedents and consequences (Nair and Vohra, 2012; Soffia et al., 2022). Seeman (1959) translates Marx' definition of alienation into five alternative social-psychological meanings (powerlessness, meaninglessness, normlessness, isolation, and self-estrangement) which focus on the perspective of the actor as distinct from antecedent structural conditions. Here he uses language of expectations, perceptions and behaviors to describe the cognitive state and subjective feelings of the actor (Seeman, 1959; Twining, 1980). This approach proved very influential, but has been criticized for a lack of focus on structural conditions shaping these alienating experiences (Blauner, 1964; Twining, 1980). In their review of literature on alienation, Nair and Vohra (2012) also note that it is not always clear whether alienation is based on one dimension (e.g., estrangement) or must be multi-dimensional as per Seeman (1959). Finally, there is also a lack of clarity around whether dimensions such as meaninglessness or powerlessness are antecedents or consequences of alienation (Nair and Vohra, 2012; Soffia et al., 2022).

Alienation—in its various guises—is fundamentally related to the socio-historical context, the social relations of work, and the self (Twining, 1980; Nair and Vohra, 2012; Soffia et al., 2022). Lukes (1977) describes alienation as referring to two key aspects: the relationship between the individual and their social environment and secondly, their state of mind. Seeking to avoid the psychologization (Godard, 2014) of the work-self relationship, and the individualization of burnout and resilience discourse, we seek a definition of alienation which can contain three key features: structural conditions, felt estrangement or disconnect with a socially-defined self, and a risk to psychological wellbeing. For the purposes of this paper we turn to Twining (1980, p. 422) definition of alienation as a social process; “…an interactional, or relational, consequence of a negative encounter of some duration which involves the degree of felt separateness from fundamental social situations in which self is being defined.” This definition posits the social-structural context as a “framework for focusing on situationally specific occurrences of alienation” (Twining, 1980, p. 422). For Twining, alienation is a structural-subjective process resulting from an individual's relational experience with their structural context, in which subjective processes and perceptions of circumstances result in feelings of disconnect between the experienced self, and a socially defined self (e.g., professional self). In concentrating on the relational nature of structure and affect, this definition offers a more dynamic frame for exploring the structural sources and emotion-management strategies of workers who experience self-estrangement, and yet continue to work. To fully capitalize on this approach, here we draw on emotion management literature, particularly Hochschild (1979), to conceptualize how workers might navigate these alienating conditions, expectations and experiences.

### Emotions as socially shaped labor and management

As our way of relating to the world around us, emotions represent an important “ever-present” in the relationship between work and the self (Hochschild, 1983; McMurray et al., 2022). Sociological and social-psychological accounts have depicted how emotions are more than just a physiological process, and are subject to norms, rules, reflexivity, regulation, labor and structural and cultural influences (Hochschild, 1979, 1983; Rosenberg, 1990; Thoits, 2004). Two points are worthy of mention here. Firstly, social structures are evident in the “feeling rules” associated with social group membership which define what is, and is not appropriate to feel in given situations (Hochschild, 1979). These “conventions of feeling” provide a reference for the fit between circumstance, expectation and feeling (Hochschild, 1979). For example, the Declaration of Geneva discussed at the outset point to the “feeling rules” of medicine as they set out the norms, expectations and practices of being a doctor, or the “rules of self-sacrifice” (Gerada, 2021). Secondly, cognition is a fundamental feature of the experience of emotions. Rosenberg emphasizes the important role of reflexivity, which involves the application of cognitive processes to physiological sensations; “…we do not simply “feel” an emotion; we also “think” an emotion” (1990, p. 5). Social structures and reflexive, cognitive processes thus shape our identification, management and display of emotions (Hochschild, 1979; Rosenberg, 1990; Thoits, 2004).

Theoretical approaches to emotion management can be categorized into three broad areas: self-regulation (i.e., private efforts of emotion work or management), interpersonal emotion management (i.e., public displays and expressions which require emotional labor to manage others' emotions), and reciprocal emotion management (i.e., simultaneous management of peers' emotions; Lively, 2000). We now briefly describe the key elements of each. In her instrumental work on emotions, feeling rules and social structure, Hochschild (1979) conceptualizes the effort it takes “to cope with feeling rules” (551). Using the terms emotion work and emotion management interchangeably, she points to individual's conscious efforts of evoking, inhibiting or shaping to manage any discrepancy between feelings experienced and what one wants to, or ought to, feel. Hochschild defines two types of cognitive emotion work; (i) *evocation* which focuses on attaining feelings that are currently absent, and (ii) *suppression* which focuses efforts on eradicating or altering unwanted present feelings Hochschild (1979). She also notes three different techniques of emotion work—cognitive (e.g., changing ideas and thoughts to change associated feelings), bodily (e.g., altering physical symptoms of emotions), and expressive (modifying expressions to change inner feelings). Importantly, these private efforts of “deep acting” are still subject to the influence of social structure via the conventions, norms and expectations set out by “feeling rules,” and may not always be successful (Hochschild, 1979; Lois, 2006; Wharton, 2009).

By concentrating on how people *try to feel* rather than how they *try to appear to feel*, Hochschild (1979) distinguishes the self-regulation, or “deep acting,” of emotion management from the interpersonal “surface acting” of emotional labor (Hochschild, 1979, 1983). Emotional labor encapsulates the second category noted above, interpersonal emotion management. Emotional labor defines the process whereby employees' private feelings and public expressions become commoditized and attain an exchange value—essentially being sold for a wage (Hochschild, 1983). Hochschild (1983) study of flight attendants illustrated the labor involved in shaping and maintaining “authentic” public displays of emotion that are aligned with capitalist feeling rules. Employees' feelings, emotions, expressions, and bodies enact this socially structured, interpersonal labor—complicating the relationship between emotions and identity. Subsequently, prolonged periods of dissonance between experienced feelings and expected emotional displays can lead to inauthenticity and self-alienation (Hochschild, 1983)—highlighting the conceptual link between alienation and emotions. Research has demonstrated links between prolonged periods of emotional dissonance and physical exhaustion, inauthenticity, alienation, depression, and psychological distress (Hochschild, 1983; Erickson and Wharton, 1997; Thoits, 2004; Erickson and Grove, 2008).

The third category of emotion management literature relates to reciprocal emotion management; defined as a support strategy used among paralegals and legal assistants to cope with work demands and stressors emanating from senior partners (Lively, 2000). Unlike the previous two categories discussed, reciprocal emotion management represents a less exploitive form of emotion management as peers turn to each other to “…engage in acts of horizontal reciprocal emotion management to alleviate their stress” (Lively, 2000, p. 33). In order to maintain professionalism, and not react “inappropriately” to senior partners' authoritarian behavior, the paralegals engaged in informal caretaking behaviors to identify shared opinions and experiences using humor and “horror stories” (Lively, 2000). However, once more highlighting the link between emotions and social structure, Lively (2000) notes how strategies of horizontal reciprocal emotional management enabled the paralegals to remain professional and deferential to partners, which served to reinforce the status hierarchy shaping interpersonal behaviors at the law firm. In supporting the paralegals to manage the emotional and interpersonal demands of their jobs, these strategies also supported the status quo, reproducing workplace stratification (Lively, 2000; Thoits, 2004).

Social structure is present in the social and cultural norms underpinning emotions as they signal the relation between our grasp of events and experiences and our prior expectations—expectations based on some form of emotional socialization (Thoits, 2004). In medicine, professional socialization is also a form of emotional socialization (Becker et al., 1961; Hafferty, 1988; Underman and Hirshfield, 2016). Through medical training doctors acquire the norms, appropriate emotion management techniques, and emotional labor requirements of the profession (Thoits, 2004; Gerada, 2021). One specific example noted in the literature is the disposition of affective neutrality, which emphasizes the valorization of objectivity, and a detached, emotionally neutral concern for patients (Smith and Kleinman, 1989; Thoits, 2004; Underman and Hirshfield, 2016). Hafferty (1988) and Smith and Kleinman (1989) demonstrated the powerful role of medical school training and culture in internalizing emotion-management rules and strategies within the clinical encounter including the detachment of fear or disgust, transforming the patient into an object, and avoiding sensitive contact. Traditionally, one of the core feeling rules of medical school is to limit empathy and remain unemotional (Underman and Hirshfield, 2016). However, empathy has now become a contested terrain within medicine with new initiatives in medical education pushing back against these practices of detachment and foregrounding the importance of “empathic healthcare” (Underman and Hirshfield, 2016; Winter et al., 2023).

Recent research has pointed to the emotion work and emotional labor required to deliver empathic care. In a study of the emotional labor employed by critical care nurses in the United Kingdom during COVID-19, McMurray et al. (2022) highlight the *empathy* of “being there” for patients and families, and the professionalism of *neutrality* in the face of public anger. Dowrick et al. (2021) also note how COVID-19 disrupted the care experience for patients and staff alike, requiring improvised care practices and the extension of emotion management tasks to ensure new ways of alleviating distress (e.g., playing the role of family member, or comforting grieving relatives). These new affective practices provided a sense of connection in the face of isolating infection control measures (Dowrick et al., 2021). Kirk et al. (2021) found “pervasive and intense” emotional labor requirements for emergency nurses who balanced a mismatch between professional values and service demands by juggling compassion, stoicism, and resentment in the face of patient frustration and grief, and doing so in the belief that this was an intrinsic part of being a good emergency care nurse. Johnson's (2015) study of private residential care home workers also notes how emotional labor illustrated a mismatch between the moral interests of residents and the economic motivations of their employer. By naturalizing their emotional labor in line with organizational strategies (e.g., unique and altruistic employees), care workers devalued their own labor while experiencing an enhanced sense of “moral righteousness” in defending the needs of their residents (Johnson, 2015).

Discussing emotional labor in medicine, Gerada (2021) emphasizes the need for almost constant attention to others' needs; “Irrespective of how we feel, or how tough a time we've had….” (Gerada, 2021, p. 24). Here, Gerada points to the strength of feeling rules that define what is expected of doctors, while also indicating a potential blurring of the line between private emotion management and public emotional labor due to the power of the “medical self” identity. For doctors, the rules defining what to feel, what to express, and how to behave (Lively, 2000) are built on the intertwining of the personal and the professional. This paper seeks to explore the emotion work required within this space between the personal and the professional by drawing on emotion management (Hochschild, 1979) and alienation (Twining, 1980) theories to understand the social structures and strategies through which doctors continue to provide care while experiencing a deteriorating relationship with their work.

## Materials and methods

### Design

We used a novel form of remote ethnography, Mobile Instant Messaging Ethnography (MIME), to explore the everyday lived experience of hospital doctors in Ireland. Using Zoom^TM^ and WhatsApp^TM^ platforms, MIME was conceived as offering a prolonged, “live,” digital connection to what had become a physically inaccessible group due to public health restrictions—hospital doctors during a pandemic. MIME involves three sequential stages of data collection built around the objectives of rapport-building, ethnographic insight, and reflexivity (Humphries et al., 2022):

1. Rapport-building: online interviews to build relationships and gather basic demographic data.
2. Ethnographic insights: a 12-week WhatsApp^TM^ conversation structured by three questions texted to participants each week with each week representing a different theme (e.g., work-life balance, supports etc.). The hospital doctors were free to respond whenever suited, they could discuss other topics where relevant, and send messages in text, voice note, or image form.
3. Reflexive conversation: online interview to reflect on, and discuss, what arose during the 12- week WhatsApp^TM^ conversation. The hospital doctors were encouraged to read through the WhatsApp^TM^ conversation prior to this interview.

The research team comprised three experienced qualitative researchers (NH, JPB, and JC) with knowledge of the working conditions of hospital doctors in Ireland through previous studies (e.g., Humphries et al., 2020; Byrne et al., 2021; Creese et al., 2021). This may have aided the initial rapport building stages of MIME engagement. However, there were no previous relationships between any researchers and participants. To ensure consistency among the researcher's approaches, each data collection stage had a semi-structured component (e.g., broad interview topic guides, and three pre-defined questions weekly via WhatsApp^TM^). While maintaining a core structure throughout, each relationship and conversation was supported to flow at its own rhythm to support the flexibility and collaborative sensemaking of MIME.

MIME is inherently a collaborative and reflexive process of data generation through which both researcher and participant help each other understand the professional life-worlds of hospital doctors (Creese et al., 2023). It uses smartphone technology and WhatsApp^TM^–both of which are used regularly by doctors (Gould, 2017; Nair et al., 2021)—to provide a flexible mechanism of instant engagement whenever, and however, was most suitable for the doctors (i.e., *in* work or *after* work). MIME advances digital diary (Berg and Düvel, 2012) and Mobile Instant Messaging Interviews (Kaufmann and Peil, 2020) approaches by replicating the constant connection of traditional ethnographic approaches via virtually “hanging out” (Ito et al., 2010) with hospital doctors to collaboratively make sense of their lived experiences. Thus, MIME is distinct in connecting researchers and interlocutors through longitudinal digital conversations eliciting details of events, interactions, and emotional observations. Additionally, this “live” connection can become an interactive, two-way digital conversation, often initiated by the interlocutor. This reduces the typical power imbalance of research participation as interlocutors can decide when to engage, and thus provide data according to their own logic, context and inclination (Creese et al., 2023).

We have written extensively elsewhere on MIME processes, rationale, the profound impact it had on the research team, and its potential for catalyzing change—for more details please see Creese et al. (2023) and Humphries et al. (2022). For the purposes of this paper, we draw *only* on the 12-week WhatsApp^TM^ conversation data outlined in step two. This represents a further development of MIME data analysis, as the long-term exchange of digital messages involved nuanced reflections on the nature, and impact, of doctors work experiences and feelings, and what was required to keep going in the job.

### Sampling and interlocutors

We used purposive and snowball sampling (June, 2021) utilizing Twitter, professional associations, the project website, and opinion pieces on digital media advertising the new study (e.g., Humphries, 2021) to reach a national audience. We also relied heavily on the use of illustration to aid our recruitment (see Humphries et al., 2022). We refer to our study participants as interlocutors throughout as they represent individuals actively involved in, and guiding, our WhatsApp^TM^ conversation. Study interlocutors self-selected based on this advertising campaign and follow-up information provided by the research team. While the pandemic-context presented a challenge to recruitment, we do not feel our sampling was impacted by challenges related to familiarity with digital devices and communication. Recent evidence shows the popularity of the WhatsApp^TM^ platform in Ireland among all age-groups (Murrell et al., 2023), and within the medical profession in particular (Gould, 2017; Nair et al., 2021). Interlocutors did not receive any compensation for participation. We recruited 28 hospital doctors to the MIME study as described in Table 1.

**Table 1:** Sample profile (*N* = 28).

**Gender**	**Female**	**20**
	Male	8
Grade	Consultant	13
	Staff Grade Specialist	1
	Specialist/Senior Registrar^*^	4
	Registrar^†^	2
	Senior House Officer (SHO)^‡^	8
Hospital location	Urban	19
	Rural/regional	9
Specialty	Anesthesiology	3
	Dermatology	1
	Diagnostic radiology	3
	Emergency medicine	2
	Internal medicine	6
	Obstetrics and gynecology	4
	Pediatrics	3
	Psychiatry	3
	Radiation and medical oncology	1
	Surgery	2
Medical degree year	Pre−1990	2
	1990–2000	4
	2001–2010	7
	2011–2020	15

^*^Advanced specialty trainee doctor.

^†^Experienced specialty (trainee or equivalent) doctor.

^‡^Basic specialty trainee (or equivalent) doctor.

The majority of interlocutors identified as female with a roughly even split between consultant and non-consultant hospital doctors (NCHDs). In the Irish health system, NCHDs are doctors who have a medical qualification but have not reached consultant or specialist grade, many of whom are on training schemes. The vast majority of doctors were located in hospitals in urban settings (e.g., three major cities in Ireland). The doctors were also working in a diverse range of specialties. Over half the sample received their medical degree post-2010 with an average of 14 years' experience as a doctor. As the grades in Table 1 also indicate, the sample provides a plethora of professional experience, important as the experience of burnout dimensions has been associated with career stage in medicine (Dyrbye et al., 2014).

### Data collection

Three authors (JPB, JC, and NH) carried out data collection from their own homes. Each researcher was responsible for taking their cohort of doctors through the three stages of MIME. While the entire data collection period ran from June—December, 2021, in effect each doctor's relationship with a researcher spanned between 14 and 20 weeks. This time period accounted for the 12-week WhatsApp^TM^ conversation, pre- and post-interviews, while also allowing flexibility for interlocutors to pause participation due to annual leave, illness, etc. This flexibility provided a sense of intimacy as the researcher was in touch while “life” happened (e.g., holidays or pandemic-related events). Twenty-six of the 28 interlocutors completed all three MIME stages. Throughout, the research team met weekly to reflect on their emotional responses to data collection and conversations with interlocutors “in the field” (Humphries et al., 2022).

This paper analyses the data generated from the 12-week WhatsApp^TM^ conversation with all 28 interlocutors. Prior to beginning this stage, interlocutors were provided with the rules of engagement detailing expectations regarding message frequency and content. This was to ensure they communicated in such a way that was comfortable for them but did not contain any references or material which may identify colleagues, patients, or any other form of sensitive data. To ensure a semi-structured approach across all conversations, we developed a 12-week topic guide based on previous research findings (Humphries et al., 2019; Byrne et al., 2021) and comprised of distinct weekly themes. Three questions were sent to interlocutors each week aligned to each respective theme. For example, the theme for week five was “belonging at work” and one of the questions asked; “Do you feel like an important part of the team at work?”

While the topic guide provided a structure to the conversations, in many cases conversations flowed in different directions depending on the experiences and feelings of the interlocutors when responding. Interlocutors were free to respond whenever was suitable, to discuss other topics, and communicate in any medium, and while a small number sent some photographs and voice notes, text message was the primary communication method. Some responded instantly to texts, while others waited until the end of the day, or week, to respond to questions—with conversations proliferating from there on a range of different issues that were relevant to them at the time of data collection. Ethical approval was received from the Royal College of Physicians of Ireland (RCPI) Research Ethics Committee in June 2021.

### Analysis

We exported all WhatsApp^TM^ conversation data to MS Word format and added this to interlocutors' interview transcripts—in effect creating a major transcript for each. All data were de-identified and all interlocutors were offered the opportunity to review their transcripts prior to input into MaxQDA for analysis. Broadly, the stages of analysis included re-reading of all transcripts to familiarize ourselves with the full dataset, co-creation of a broad coding schema, indexing and allocating data to these broad codes, and finally inductively exploring the coded data for themes and patterns across the dataset (Dey, 1993). As such, deductive and inductive coding processes were utilized. JPB indexed the full dataset to a set of codes established by the research team (NH, JPB, and JC). Codes included “voice,” “working hours,” and “support structures.” These codes also included the exhaustion, detachment and inefficacy dimensions of burnout. All authors discussed the conceptualization of the findings and the illustrative data employed.

Abductive principles (Tavory and Timmermans, 2014) inspired the analytical process for this paper. Abduction refers to a pragmatic, recursive and iterative analytical process of theory construction founded on surprising evidence, revisiting of data, and acknowledgment of the authors' intellectual background (Timmermans and Tavory, 2012; Tavory and Timmermans, 2014). The initial analysis focused on the antecedents and experience of burnout dimensions for hospital doctors. However, on deeper analysis, JPB identified a *key surprise* within the data as interlocutors, in recounting their experience and reflecting on their subsequent feelings, often spoke to the emotions and emotion work required to manage a profound disconnect from larger social structures shaping their working life (e.g., the hospital, their profession, and the public). Drawing on his background in the sociology of work, JPB then *revisited the data* to focus specifically on the emotion-management and alienation within the data. This involved re-analyzing the sub-set of MIME data indexed to burnout dimensions to explore how it may be alienation underpinning these experiences. This necessitated a shift in focus from individual experiences of burnout to the role of social, professional, and organizational structures in shaping feelings of disconnect and the emotion-management requirements of hospital doctors. To ensure confidentiality and aid the flow of the narratives described, we have given each interlocutor a pseudonym. Table 2 provides an overview of the pseudonyms and demographics for the 18 interlocutors referenced in the results. We have kept emoji's in statements to stay true to interlocutors' intonation and meaning in text messages.

**Table 2:** Interlocutor pseudonyms (*N* = 18).

**Name**	**Gender**	**Grade**	**Location**
Amanda	Female	Consultant	Urban
Grace	Female	Consultant	Urban
Peter	Male	Consultant	Regional
Gemma	Female	SHO	Urban
Lisa	Female	SHO	Urban
Barbara	Female	Consultant	Regional
Sara	Female	Consultant	Urban
Henry	Male	SHO	Regional
Della	Female	SHO	Urban
Laura	Female	Consultant	Urban
George	Male	Consultant	Urban
Joanne	Female	Consultant	Urban
Oliver	Male	Consultant	Regional
Joshua	Male	SHO	Urban
Zoe	Female	Consultant	Urban
Chloe	Female	SpR	Regional
Thomas	Male	SHO	Urban
Lily	Female	SpR	Regional

### Limitations

There is potential for self-selection bias as interlocutors may represent those who wanted to complain about their experience in the Irish health system. In light of the pandemic context, recruitment of interlocutors was more challenging than normal. The sample does not contain any non-training scheme NCHDs. Due to the MIME design, we did not gain direct insight into, or data from, the physical, environmental, and cultural context of the hospital as a workplace, or the interactions between doctors and colleagues—as a traditional ethnography would have done (Kietlińska, 2022). Instead, MIME relies on the engagement of participants within the digital conversation to generate data. Nonetheless we did get access to a wide range of working lives nationwide with a focus on the emotional and cognitive reflection on work (i.e., emotion-management). Finally, there are no interns or non-trainee doctors in the sample.

## Results

*As a doctor on a busy shift, I occasionally feel like I'm the enemy because I can't provide what patients need* (Lisa).

*I saw a great poster in ED about tips for coping with stress at work where someone had scrawled—“the lashings will continue until morale improves!” underneath and it's so true* (Joshua).

Drawing on alienation and emotion management literature to explore the experiences of hospital doctors' deteriorating relationship with work, we delineate two types of emotion management strategy used to suppress undesired feelings and emotions—acquiescence and depersonalization. The hospital doctors drew on cognitive and behavioral techniques to suppress feelings of powerlessness, frustration and guilt, and continue to provide care. As the two quotes here exemplify, these strategies were necessary to negotiate the disconnect between professional norms of expertise and self-sacrifice, and organizational realities of impotence and self-preservation—all of which challenged doctors' sense of professional self and sense of autonomy—key psychosocial processes of alienation. We now discuss each suppression strategy type in turn.

### Negotiating expertise and impotence: acquiescence as suppression strategy

A number of doctors pointed to the frustrating nature of working within an under-resourced, inefficient and dysfunctional health system where working tirelessly often resulted in minimal influence. Gemma described how, over the last week, she was “just feeling a bit impotent” as she didn't seem to be able to provide the necessary care to many presenting patients; “…what we have to offer to people seems a bit rubbish.” Joshua noted the “sheer chaos” and endemic inefficiencies of the postgraduate training pathways which, rather than providing any sense of efficiency and satisfaction, often require “…grinding ourselves into nothing.” This phrase perfectly captures the enervating nature of negotiating professional expertise and organizational impotence. To manage the negative feelings emerging from these conditions, the doctors employed cognitive emotion-management techniques resulting in a form begrudging acceptance, which we term acquiescence.

The doctors' accounts described highly skilled professionals who rarely had the requisite resources to ensure the requisite care. Throughout our virtual conversations, the doctors noted the rarity of “…being able to do what needs to be done” (Amanda) for patients due to resource deficits and dysfunctional systems. Many described a sense of obstructed autonomy and its impact, like Barbara:

…*less interested in Medicine as a career. Trying to find alternatives…. I think the demands are becoming intolerable when the resources are so deficient- it makes it harder to do the job properly as one is trained to do…. …no investment in infrastructural deficits—makes the job unnecessarily stressful, busy and ultimately energy sapping and depressing…. after repetitive blank walls one becomes disillusioned…. we are capable of so much more!!!* 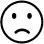 (Barbara).

Here Barbara describes her inability to utilize her training and expertise to influence patient care due to severe resource deficits. Consequently, she notes an alienating disconnect between the knowledge of care required and the nature of care provided (“capable of so much more”), which brings together professional training, organizational deficits and strained work conditions. Barbara noted that she had begun to focus her thoughts on re-framing her future, even potentially outside medicine to achieve a better work-life balance. This points to a cognitive strategy of focusing thoughts on an alternative, positive future venture to suppress the strain of present conditions.

When asked how he felt at work today, Thomas, an SHO working on a surgical rotation in a busy urban hospital, provided a powerful example of the impact of the disconnect between professional expectations and organizational realities on doctors' emotional and psychological wellbeing. Thomas described how, that day, he had turned away a number of patients, some of whom had waited months and years for surgery, because the service was so understaffed and there was no consultant in the clinic. As he lacked the expertise for some of the more complex cases, he had to tell these patients to return at another time. Thomas noted that the patients, while upset and frustrated, were mostly “…disappointed that the system was failing us both.” That evening, Thomas described how that experience had made him feel.

*Terrible, guilt ridden and under pressure…. It felt terribly inappropriate and yet wasn't anyone there's fault. I felt angry and resentful that these people were not being provided with doctors to care for them…. This job…is so intense…I just feel in survival mode all the time so don't give as much thought to how I'm coping or feeling other than for these 3 texts a week* (Thomas).

For many of our interlocutors, these experiences represent more than just the inefficacy associated with burnout as they point to the emotional impact of a mismatch between professional feeling rules and experienced feelings (Hochschild, 1979). Thomas' experience highlights the interrelation of social-psychological (e.g., powerlessness) and structural (organizational deficits) elements of alienating conditions (Twining, 1980) which serve to disconnect doctors from their professional expectations, expertise, their patients, and themselves. To cope with these alienating conditions and feelings, Thomas notes how he is in “survival mode,” which requires ignoring how he is feeling—apart from when the researcher inquired! Here, “not thinking” represents a conscious cognitive emotion-management strategy of suppression. Much like Barbara previously, this involves a reframing of cognitive focus away from himself, and the present; “it's a case of head down and get it over with” (Thomas). Thomas also described feeling undervalued and under-utilized as his abilities and training were not being used effectively—in a system that urgently needed them. In his case this led to feelings of guilt, anger, and disappointment; “…I constantly feel like I am letting my team, my patients, my family, and friends down…” (Thomas).

Laura, a consultant in an urban hospital, described helplessness as an adaptation made by both staff and patients in order to endure the deficits of the health system in Ireland. Recalling the introductory quote from Samuel Shem's *House of God*, Laura noted how this cognitive adaptation had become “a survival instinct” so that you continue to do your best while *accepting* that “nothing can be done to fix things.” When Laura's researcher described a previous message from her as reading like a war zone, she picked up and expanded this metaphor, observing:

…*even if you don't see yourself as being in the trenches, it is kind of like being in a never ending war situation with rationing and not enough supplies. You just accept that all you can have is tinned spam instead of fresh food and try not to think about it from day to day. Except then occasionally you explode!!* (Laura).

By “trying not to think about it,” Laura articulates a conscious, cognitive emotion management strategy for suppressing undesired feelings and negotiating expertise and impotence. Using war-time analogies, she notes a cognitive shift from unease to acceptance of rationed care provision—even knowing that it may not be the standard it should be. Accepting little can be done and trying not to think about it, became ways through these conflicting daily conditions. Laura's depiction highlights the alienating effect of unmet expectations around professional expertise as doctors employed this strategy of normalized helplessness and apathy. Responding to a query about morale in his workplace, George described the “general lassitude around the place” due to how difficult it was to get a good day's work done, noting that it was the exception rather than the rule. Importantly, he also warned how people will stop caring or pushing for improvement and this will be difficult to reverse. Similarly, other interlocutors noted becoming accustomed to “always delivering poorer care than I want to” (Joanne) and tolerating “less than is good” (Sara).

Acquiescence, doctors' attempts at suppressing frustration, anger, and helplessness and accepting the status quo to ensure some form of sustainability, is a response to alienating conditions that pit specialized expertise and skills against an organizational context which inhibits their use; “…you accept the flawed system…. we're all a bit broken by it” (Lily). The interlocutors employed cognitive emotion management techniques to reframe and refocus their thoughts away from their present conditions, and from themselves—to endure these alienating conditions and avoid “exploding.” The divergence of professional expectations and organizational realities became even more pronounced when interlocutors discussed the need to juggle patient care with care for themselves.

### Negotiating sacrifice and self-preservation: depersonalization as suppression strategy

The doctors' narratives described the context and demands of a “greedy profession” (Coser, 1974; Humphries et al., 2020) that continually demands more of their working lives, limited organizational supports, and the subsequent need to secure their own methods of self-care. To suppress negative feelings emerging from the disconnect between professional norms of self-sacrifice and personal imperatives of self-care and self-preservation, the doctors turned to cognitive and behavioral techniques which resulted in a state of detachment and reduced empathy which we categorize as depersonalization.

Chatting remotely one evening about the healthcare heroes narrative popularized during the COVID-19 pandemic, Chloe sent on an illustration of a doctor with angel wings holding up the globe, noting this “…stuff is going to come back to bite us.” Probing this further it seemed the religious calling undertones of the narrative really bothered Chloe as they implied there was no need to look at improving doctors' working conditions because everyone is so “…impressed about how medical staff were such Good People [caps in original message] who loved making personal sacrifices.” Chloe's irritation with this illustration indicates a frustration with the conflicting sentiments within the Declaration of Geneva that ask doctors to dedicate their lives to service *and* attend to their own health. She felt these narratives reinforced public and professional norms around doctors not requiring the same conditions and supports as normal people. In the face of organizational abandonment, the interlocutors turned to each other, and their own wellbeing.

Indicating the importance of reciprocal forms of emotion management (Lively, 2000), Zoe makes a distinction between informal peer support and organizational initiatives. She described peer support (coffee or lunch debriefs) as very important and usually available, while organizational supports (e.g., pilates classes) are only available for those who work from nine-to-five, and therefore of very little relevance to “…those in the hospital who need them most.” Zoe goes on to note the unintended consequences of such approaches to wellbeing supports; “I feel disconnected though at times from the rest of the hospital.” Other interlocutors noted the “tone deaf” nature of initiatives sent by email and held between nine and five—when they did not have time to read emails, never mind attend the sessions. Chloe took a more cynical approach in describing the “naggy emails to come to wellness seminars” held at inappropriate times so that “we can learn how not to be burnt out.” Peter felt that it was “mainly up to me to look out for myself… Managers haven't shown much interest.” Under the weight of a demanding profession, public expectations, and organizational support- and resource-deficits—all of which could consume their time and energy completely—Thomas described the need to individually find a balance between personal sacrifice and self-preservation.

*I also have noticed more and more that my practice is mostly aimed at self-preservation, I will engage less with patients, my empathy and compassion have worn away and I think constantly about getting a chance to get away to eat and/or sleep and those are my main priorities at work rather than patient care, I feel really guilty saying it but unfortunately I realize that I won't be able to provide any care at all if those aren't my priority…* (Thomas).

Discussing how he felt at work that day, Thomas encapsulates the need to find a strategy to balance self-care and patient care that echoed through many of the doctors' narratives. In reflecting on how he was “engaging less with patients,” Thomas describes a behavioral strategy (Hochschild, 1979; Rosenberg, 1990) which enables him to suppress feelings of guilt, and subsequently experience a reduction in empathy and compassion for patients—violating a key feeling rule of medicine which must now be managed. This strategy is in order to ensure he can continue to provide any care at all—implying the potential negative impact on his wellbeing should he not prioritize his own needs. This example represents more than a practice of detached concern, or affective neutrality. Strikingly, his strategy is the result of a perceived choice between self-preservation via suppression of personal guilt and patient empathy, and personal sacrifice at the cost of physical, mental and emotional wellbeing. Guilt was a common sentiment expressed by the interlocutors, representing the manifestation of the disconnect between professional ideals and survival requirements. Grace described how she often felt like “…the [health] service runs on good will and our guilt that we might let our colleagues or patients down.” Like Thomas, Grace also noted the delicate balance of self- and patient care stating that she “could do with caring less to protect myself.” Interestingly, here Grace acknowledges and reflects on the importance of depersonalization strategies of “caring less” to suppress feelings of guilt and protect her own wellbeing, but admits she has been unsuccessful at carrying this out. Emotion management, even when unsuccessful (Hochschild, 1979), still takes a toll; “I have felt very close to that [burnout] recently” (Grace).

Equally striking in Thomas' account is the basic nature of the needs that must be met (e.g., food and rest) at the cost of empathy and compassion for patients. Violating the complementarity of the Declaration of Geneva statements, Thomas almost depicts a contest between his professional identity and his bodily health. This contest between professional ideals and basic physical needs was crystallized by Henry who commented that he had recently noticed that he had become so accustomed to fasting during (extended) working hours that he was not really eating much during his week off work. Joshua recalled that after a recent call shift he drove straight to a petrol station to buy “about 3 l of fluid to just hydrate, it's kind of crazy.” Eliciting more wartime analogies, Della highlighted the impact on her physical wellbeing and the importance of the camaraderie provided by peers facing the same extreme conditions: “[I] don't eat properly or exercise because I spend all my time in work… It's only the fact that a lot of the team are the same and we get so tired together we end up crying laughing at hour 14 of the day that makes it any way survivable.” Here Della points to the reciprocal emotion management of support from peers who are suffering similarly to help manage the impact of the work demands and suppress, or at least alleviate, the impact of the job on her diet and fitness. Shared experiences and dark humor among peers seem to help Della detach from her own bodily needs to ensure she can manage the physical and mental strain of the role. Finally, the doctors also described the impact of failed emotion management due to extreme fatigue leading to an inability to ensure empathy for patients and families.

Aligned with the common feeling of guilt, the doctors described a struggle to maintain consistent levels of empathy in the face of physical and mental fatigue. Like Thomas, Della stated that; “We're all struggling to be empathic and well-rounded by now.” Here, Della notes the failure of both deep acting and surface acting (Hochschild, 1979) with interlocutors unable to manage their feelings or the expressions and behaviors that follow. Oliver, a consultant working in a regional hospital noted how really long shifts led to intense tiredness and the thinning of patience. Highlighting once more the divergence of professional ideals and organizational demands, he noted that during calls with staff he was “…just slamming the phone down to be honest, not very professional, at that level of fatigue you lose all inhibitions and become quite abrupt.” Chloe added that the issue is not just tiredness, rather “…you get really impatient and really blunt long before that…” leading to reduced empathy and negatively perceived communication styles. Recalling a specific incident, Chloe described how she held a conversation with a patient's family to discuss the prognosis. She felt the conversation had gone ok, despite the family being upset at the prognosis. However, the family felt that Chloe had been extremely blunt, and made a complaint. “I had literally not slept in 40 h so I probably was blunt… you think you're used to it and you might remember to say the right thing but it can be obvious to the patients that you're going through the motions of sympathy.” Here extreme fatigue leads to an inability to manage, control and predict feelings and expressions—rather than an enhancement of an ability to practice affective neutrality (Underman and Hirshfield, 2016). This example highlights another form detachment—between how the doctors try to feel (e.g., empathetic, patient) and how they “try to appear to feel” (Hochschild, 1979, p. 560). Due to extreme working conditions, their inability to “evoke” the required feelings leads to a failed attempt at displaying appropriate feelings. The survival mode of self-preservation required of the doctors to endure extreme tiredness and hunger can come at a cost of self-regulation abilities, and subsequently the quality of care.

Depersonalization, interlocutors' efforts at suppressing feelings of guilt, abandonment and poor health, represent a strategy for negotiating a structural context (professional norms, organizational deficits, and public expectations) which requires doctors to risk impairing their own health, or the quality of their care. Cognitive and behavioral techniques for negotiating the balance between personal-life sacrifice and self-preservation included engaging less with patients, reprioritizing wellbeing, and seeking peer support. The doctors' accounts also describe incidents of failed emotion management where they became detached from their own feelings, emotions and behaviors due to extreme fatigue. Doctors' sense of professional self was challenged by an erosion of empathy for patients alongside a re-prioritization of their own wellbeing needs, like Joanne who was “trying to be a bit compassionate toward myself.” This depersonalization reflects a sense of self caught between professional ideals of high-quality care, and self-preservation so as to provide any care at all.

## Discussion

*Institutional rules run deep but so does the self that struggles with and against them* (Hochschild, 1983, p. 229).

This paper contributes to medical sociology and sociology of emotion literature by integrating emotion management and alienation perspectives to delineate the strategies of acquiescence and depersonalization used by hospital doctors to manage experiences and emotions that violated the feeling rules (Hochschild, 1979) and professional norms (Gerada, 2021) of medicine. *Acquiescence* was characterized by cognitive techniques that refocused thoughts away from the present conditions to future alternatives, and away from the self. This technique served to suppress feelings of anger, frustration, and powerlessness emerging from experiences of obstructed autonomy, resource deficits and inferior care. *Depersonalization* reflected cognitive and behavioral techniques of withdrawing engagement with patients, limiting empathy, and seeking peer support, to suppress feelings of guilt and abandonment and cope with the impact of the job on their physical and mental health. Depersonalization was also evident in examples of failed emotion management where doctors acknowledged becoming detached from their own feelings, emotions and behaviors due to extreme fatigue. Highlighting the self-estrangement at the heart of this work-self relationship, both acquiescence and depersonalization strategies involved a shift of focus away from the present self—cognitively and physically—to withstand their working conditions.

This paper advances emotion management (Hochschild, 1979; Rosenberg, 1990; Lively, 2000; Lois, 2006; Dowrick et al., 2021), alienation (Lukes, 1977; Twining, 1980), and medical identity (Gerada, 2021) literature in depicting how the socio-cultural ideals of the medical profession (healer, self-sacrifice, authority) are experienced through the expectations, emotion-management, and professional self-estrangement of hospital doctors. Building on the work of Braithwaite et al. (2017), the doctors negotiated a disjuncture between *work-as-professionally-imagined* and *work-as-organizationally-done*. The results illustrate how institutional norms and expectations do “run deep,” and are often unrealized, with the subsequent disconnects powerfully influencing doctors' relationship with work. A relationship that is really a story of disconnect: between knowledge and practice; between an idealized and actual professional self, and; between self-care and patient care. Negotiating these disconnects challenged doctors' sense of professional self and sense of autonomy.

The results illustrate the difficulty of realizing the Physician's Pledge (World Medical Association, 2017) when they are embedded in a real-world context of diverging demands and resources, in particular regarding the two recent additions. The first refers to the sharing of knowledge and expertise to benefit the patient and advance healthcare. The doctors in our study recounted numerous instances where they did not have the required resources to provide what the patient needed, despite tireless efforts and long working hours. Adding to findings from Johnson (2015) and Kirk et al. (2021) on the experienced mismatch between professional and organizational values, and service demands, the results depict feelings of anger, frustration and powerlessness stemming from this repeated disconnect between knowledge and practice. Following Chaudhry et al. (2021), acquiescence, for our study interlocutors, represented a conscious strategy of sustainability, or survival, to minimize the adverse health risks of prolonged resistance. The cognitive techniques (e.g., reframing and refocusing to shift concentration away from present conditions and self) used to manage these experiences of eroded autonomy provide a real-world reflection of the professional advice described in Samuel Shem's fictional satire at the outset of this paper.

The second addition to the World Medical Association (2017) relates to doctors attending to their own health and wellbeing to provide high-quality care. For the doctors in our study, self-care and patient care was a *choice* rather than a *complementarity*—highlighting the contradiction at the heart of this pledge. Going further, they noted the contest between ensuring the basics of their own self-care to provide *any care* at all, and fully engaged, empathetic patient care. Supporting Gerada (2021), many interlocutors noted the personal sacrifices required in medicine in describing the choice between prioritizing the self and associated guilt, or risking their own wellbeing to focus all their energies on patients at all times. To suppress these feelings of guilt the doctors drew on cognitive and behavioral management techniques of depersonalization i.e., engagement withdrawal and a conscious, and unconscious, reduction of empathy to re-prioritize their own wellbeing. Research has demonstrated the association between empathy and patients' perceived quality of care (Derksen et al., 2013). McMurray et al.'s (2022) study of emergency care nurses highlights the importance they attached to “being there” for patients during COVID-19. Yet, this study points to alienating conditions in which the reduction of empathy became a natural consequence of understaffing, long hours, and extreme tiredness. Additionally, the results indicate how feelings of organizational abandonment may play a key role in experience of alienation. To suppress feelings of abandonment and manage the physical and mental strain of their roles, the doctors turned to each other for support. Supporting Lively (2000) and Dowrick et al. (2021), the results point to the role of reciprocal emotion management in the form of regular coffees, debriefs and caustic humor (Hafferty, 1988) to assist with refocusing thoughts away from the physical and mental impact of their extreme working conditions.

Three further points on the depersonalization strategy are worthy of mention here. Firstly, highlighting the limits of seeing alienation solely through a social-psychological perspective (Seeman, 1959), the results depict depersonalization as a driver of emotion-management strategy (Lois, 2006; Wharton, 2009; Chaudhry et al., 2021) rather than a consequence—as doctors disconnect to survive. Secondly, this emotion-management strategy essentially involved the doctors bringing their own physical needs and body into focus. Here the doctors' bodies themselves become a site of contestation between professional expectations and their own physical, emotional, and mental wellbeing. Contravening the notion that physician self-care can assist in improving patient care (Parsa-Parsi, 2017), the results point to a choice of impairment: their own health, or their sense of care provision. Finally, Underman and Hirshfield (2016) note a shift in medical education emphasis from practices of detached affective neutrality to more empathetic styles. However, our results indicate a detachment from the job entirely rather than the patient within the clinical encounter. These alienating conditions and strategies may undo attempts in medical education and training to improve empathy with patients at the bedside (Winter et al., 2023). Structural conditions in which one must limit their empathy to continue to provide care completely undermine attempts to improve empathy as a key care-provider skill (Underman and Hirshfield, 2016; McMurray et al., 2022).

Highlighting the continued relevance of social theories of emotion management (Hochschild, 1979) and alienation (Twining, 1980), the results illustrate how the doctors navigated the alienated space between professional socialized expectations and responses to organizational reality and demands by trying to reconcile what they felt, with what they ought to feel. The doctors' deteriorating relationship with work is one defined by a socially shaped alienation (Twining, 1980) as they describe failing to meet professional, personal, organizational, or public expectations resulting in a feeling of disconnect from what they are supposed to *do*, and *be* (Gerada, 2021). Here, our results indicate the potential for blurring between emotion work and emotional labor due to the power of the “medical self” (Gerada, 2021). For those whose work is a vocation (a way of life), there is an intrinsic overlap of the personal and professional such that the distinction between the use and exchange value of performances is blurred. The inability to shed the medical self and the associated rules that determine “what is ‘due' in each relation, each role” (Hochschild, 1983, p. 18) in so far as doctors must always meet the “call” for help, means that they rarely have sole discretion of performances offered. An inappropriate emotional performance, in or out of work, risks damage to the personal-professional reputation, and as such doctors' emotion management readily straddles the boundaries of use and exchange (Hochschild, 1983).

The paper shows how a sense of usefulness at work is shaped by the social relations underpinning the work experience—even for doctors (Soffia et al., 2022). Following Berardi (2009), these doctors represent an alienated form of autonomy, in that their work is primarily cognitive, autonomous, and high-skilled, yet they still find their experience of work as externalized (e.g., just to keep the system running rather than provide high-quality care) and feel that their agency is constrained (Zuger, 2004). These experiences of self-estrangement and constrained agency to use their expertise fully to meet their professional identity represent alienating social structures (Twining, 1980). This raises an important sociological point on the link between emotion management and social structure (Hochschild, 1979). The strategies described in the results, when successful, enable the hospital doctors to manage their work-self relationship, while also enabling the reproduction of problematic (i.e., alienating) work structures and professional cultures. As such, emotion management strategies may help them to continue to provide care, while reproducing the very conditions that require them. Kirk et al. (2021) also note how the conditions of emergency care can heighten the need for emotional labor while also engendering the problematic feeling rules of professional cultures. In the context of our study, successful emotion management may obscure the true impact of deteriorating work environments and work-self relationships, as well as under-resourced systems, on the quality of healthcare provision.

At a practical level, the deterioration of hospital doctors' relationship with work is a threat to health systems globally—only intensified by COVID-19. Academic and policy literature has identified two important points: medicine is in the midst of a burnout crisis (National Academies of Sciences Engineering and Medicine, 2019), and doctors' working lives and wellbeing are fundamental to the sustainability of healthcare systems (Byrne et al., 2021; Creese et al., 2021). This is despite the development of a vast array of interventions aimed at supporting doctors' mental wellbeing, which have tended to target methods for improving resilience, or how individual doctors can cope with their organizational environments (Kinman and Teoh, 2018; Byrne et al., 2023). The hospital doctors highlighted the inappropriateness of information and timing regarding various wellbeing interventions, only serving to reinforce a sense of disconnect from the organization. To address this epidemic of distress, dissatisfaction and burnout among doctors, this paper conveys the need to get to the core of the issue: doctors' deteriorating relationship with work.

We need to move beyond psychologized frames of the problem (burnout), experience (exhaustion) and solution (resilience). Using alienation broadens the frame of analysis to identify how doctors' relationship with their work is shaped by professional norms and organizational realities, and the complex impact these structures have on doctors' everyday lives. Understanding *how* doctors and healthcare providers manage these conditions provides crucial intelligence for healthcare professions and organizations to appreciate how alienation continues to operate within today's healthcare systems and the impact it has on staff wellbeing, retention, and the quality of care. The alienation frame also points to the limited sphere of influence for self-care resources such as mindfulness or resilience training in the face of structural dynamics that result in the need for emotion-management strategies. Here, the onus is on healthcare systems and organizations to minimize feelings of abandonment by aligning wellbeing needs and supports so that they are relevant and available for those who need them most. The findings highlight the importance of addressing the social structures at the root of this deteriorating relationship and the broad futility of self-care interventions that are embedded in a context of unrealized professional ideals, organizational resource deficits and unhappy doctors, patients and families.

## Conclusion

Doctors' deteriorating relationship with work is a threat to healthcare organizations and health systems globally. This complex relationship is about more than burnout, or individual responses to stress. This study highlights the role of professional expectations and organizational realities that present hospital doctors with experiences of daily disconnect and the need to employ emotion-management strategies through which they can *continue to provide care* in the face of severe resource-constraints and alienating conditions. Unpacking doctors' deteriorating relationship with work requires an unpacking of the institutional context in which it is embedded, to improve the alignment between professional expectations and organizational realities. This broadening of perspective is crucial to avoid the reproduction of medical working contexts that ask doctors to choose between their own health and the quality of their care.

## Data availability statement

The dataset generated and analyzed during this study are not publicly available due to privacy/confidentiality concerns. Reasonable requests for access can be made to the corresponding author who will consider any such requests in collaboration with the project PI and the RCPI Research Ethics Committee. Requests to access the datasets should be directed to NH, nhumphries@rcsi.ie.

## Ethics statement

This study involving humans was approved by the Royal College of Physicians of Ireland (RCPI) Research Ethics Committee. The studies were conducted in accordance with the local legislation and institutional requirements. The participants provided their written informed consent to participate in this study.

## Author contributions

NH obtained funding for the study. NH, J-PB, and JC designed the MIME approach and carried out data collection. J-PB coded data collected, led the conceptual analysis and design for this paper, and led the writing team. All authors contributed to manuscript design and revision and read and approved the submitted version.
